# Irrigation scheduling for potatoes (*Solanum tuberosum* L.) under drip irrigation in an arid region using AquaCrop model

**DOI:** 10.3389/fpls.2023.1242074

**Published:** 2023-09-26

**Authors:** Juan Yin, Yingpan Yang, Rasu Eeswaran, Zhen Yang, Zhenghu Ma, Fubin Sun

**Affiliations:** ^1^ School of Civil and Water Engineering, Ningxia University, Yinchuan, Ningxia, China; ^2^ Engineering Research Center for Efficient Utilization of Water Resources in Modern Agriculture in Arid Regions, Yinchuan, China; ^3^ Ningxia Research Center of Technology on Water-saving Irrigation and Water Resources Regulation, Yinchuan, China; ^4^ Department of Plant, Soil and Microbial Sciences, Michigan State University, East Lansing, MI, United States; ^5^ Department of Agronomy, Faculty of Agriculture, University of Jaffna, Kilinochchi, Sri Lanka

**Keywords:** canopy cover, crop modeling, biomass, water use efficiency, crop water requirement

## Abstract

Potato is one of the key food crops and China is the largest potato producer in the world. However water scarcity is the major constraint to increase the productivity of potato in the arid regions such as Ningxia in northwest China where this crop is extensively cultivated. The overall objective of this study was to optimize the irrigation for potato cultivated under the drip irrigation. To do this, the AquaCrop model was calibrated and validated using the data obtained from two years of field experiment. Then, the calibrated crop model was used to simulate growth and tuber yield of potato in response to 30 different irrigation schemes under two different irrigation scenarios. The crop model evaluation parameters namely, the root mean square error (*RMSE*), the index of agreement (*d*), the normalized root mean square error (*NRMSE*) and the coefficient of determination (*R^2^*) showed that the AquaCrop model could simulate the growth and yield of potato under the drip irrigation with different irrigation treatments with reasonable accuracy. Furthermore, yield of potato has increased with increasing amount of total irrigation under drip irrigation; however, yield begins to decline when the amount of total irrigation exceeds 2500 m^3^ ha^-1^. The study also found that the optimum irrigation schedule for potato was 20 mm of irrigation quota at 7 days of irrigation cycle (i.e., 1800 m^3^ ha^-1^ or 180 mm of total irrigation). The above irrigation scheduling has achieved 46.77 t ha^-1^ of tuber yield with 15.74 kg m^-3^ of water use efficiency. These findings may be evaluated in potato cultivation across different climate and soil conditions for wide applicability at different arid regions of the world.

## 1 Introduction

China’s Ningxia Hui Autonomous Region is one of the arid regions in the world, where water resources are very limited and socio-economic development is almost dependent on the effective supply of water from the transiting Yellow River (Yao et al., 2020), with water scarcity and drought being the main challenges for potato production in the region. The potato is the fourth largest food crop in the world after maize, rice and wheat, in the order of extent of the area planted (Tang et al., 2018). Furthermore, China is world’s leading potato producer with more than 90 million metric tonnes of potatoes produced from 4.8 million hectares in 2018 (Wang et al., 2023). Meanwhile, different irrigation quotas and irrigation frequencies have a significant effect on the growth and yield of potato (Yang et al., 2023). Therefore, the development of a reasonable irrigation regime and the efficient use of limited water resources (Sammis et al., 2012) is of great importance to the agricultural development of the arid Ningxia region.

The AquaCrop model is one of the widely used process-based crop models and can simulate biomass production and yields of crops, especially in response to water (Abedinpour et al., 2012). Compared with other crop simulation models, the AquaCrop model has the advantages namely, requiring of fewer input parameters, wide applicability, simple user interface, intuitiveness, and high accuracy. The model simulates crop yield through crop canopy cover and harvest index under different agronomic management and irrigation patterns (Shirazi et al., 2021). Currently, researchers from the United States, Canada and Syria have conducted substantial amount of research on localized debugging and validation of AquaCrop model parameters, with models mainly on wheat (Jalil et al., 2020), maize (He et al., 2021) and rice (Kim et al., 2021).

There are studies on simulating the growth and yield of potato in other regions, but fewer studies have been reported on the optimization of irrigation for potato under the drip irrigation in the arid regions such as Ningxia in China, where supplemental irrigation plays a huge role in determining crop productivity. Therefore, the objectives of this study are 1) to investigate the applicability of the AquaCrop model to simulate growth and yield of potatoes grown under drip irrigation, 2) to examine the effects of different irrigation quota and irrigation cycles on the yield and water productivity of potatoes, and 3) to determine the optimal irrigation regime to increase the yield and water productivity of potatoes grown under drip irrigation in an arid environment.

## 2 Materials and methods

The methodology involved in this research study is sequentially described in this section.

### 2.1 Experimental location

The experimental site was located in Concentric County, Wuzhong City, Ningxia (36°48′2″N; 106°21′53″E, altitude 1489 m, *amsl*), a typical area in the central arid zone of Ningxia. The study area has a continental arid climate, with a large temperature difference between day and night, year-around drought and little rainfall, long daytime and strong surface evaporation, with an average multi-year rainfall around 270 mm and an annual evaporation of about 2325 mm. The soil type was sandy loam and the soil properties in the tillage layer of the experimental site before sowing were as follows: total salt of 0.6 g kg^-1^, organic matter of 6.65 g/kg^-1^, alkaline nitrogen of 38 mg/kg^-1^, fast-acting phosphorus of 3.94 mg kg^-1^, fast-acting potassium of 130 mg kg^-1^, total nitrogen of 0.027%, total phosphorus of 0.064% and 1.74% of total potassium.

### 2.2 Experimental design

The field experiment was conducted for two cropping seasons, from April 2019 to October 2020. The field was found with less variability in slope and other uncontrolled variables. Therefore, the experiment was designed as a single-factor completely randomized field experiment with six treatments and three replications of varying irrigation quota under drip irrigation for the period of two years, The trial is repeated in the second year (i.e., 2020) at the same trial site and the irrigation rates were adjusted according to the first year’s trial referring to the “Ningxia Potato Drip Irrigation Planting Technology Regulations” issued by the Water Resources Department of Ningxia Hui Autonomous Region in 2017 (Ningxia Water Resources Department, 2017). The details of the experiment and the treatments are shown in **Table 1**, and the amount of irrigation applied for each treatment at different growth stages of potato is presented in **Table 2**.

**Table 1:** Details of the field experiments and treatments.

Year	Treatment	Sowing date	Start of irrigation	End of irrigation	Number of irrigations	Irrigation amount (mm)
2019	T1	April 30	May 18	August 26	9	90
	T2					150
	T3					210
2020	T4	May 4	May 18	August 26	10	90
	T5					135
	T6					180

**Table 2:** Number of irrigations applied at different growth stages of potato during the experimental years of 2019 and 2020.

Year	Potato growth stages					Total
	Bud growth stage (May 10 – June 05)	Seedling stage (June 06 – June 25)	Tuber formation stage (June 26 – July 25)	Tuber growth stage (July 26–August 20)	Starch accumulation stage (August 21–September 27)	
2019	9% (1)	16% (2)	25% (3)	50% (3)	0	100%
2020	8.85% (1)	17.7% (2)	31.86% (3)	32.74% (3)	8.85% (1)	100%

Duration of potato growth stages and number of irrigations applied at each growth stage are given in parentheses.

**Figure 1:**
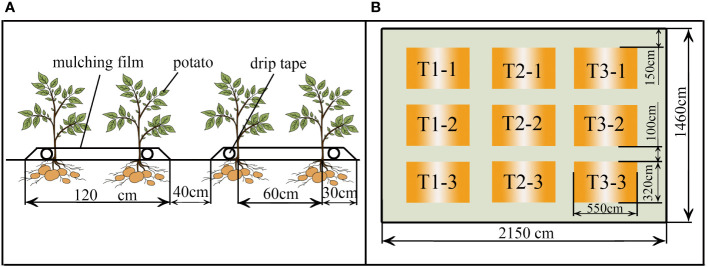
The arrangement of potato crops **(A)** in this field experiment and the experimental layout of the field trial **(B)**.

The potato crops were planted at an inter-row spacing of 60 cm and intra-row spacing of 50 cm, with each row planted with 10 plants and each plot planted with 40 potato plants, with a potato planting density of 33,345 plants ha^-1^, as explained in **Figure 1A**. The test plots were all 17.6 m^2^ in size, 5.5 m long and 3.2 m wide, surrounded by protected rows of 1 m wide between plots and 4.5 m wide at the periphery, with three replications of nine plots for each treatment in the two-year trial, as shown in **Figure 1B**.

The drip irrigation pipe was embedded to supply the water with an inner diameter of 16mm, a wall thickness of 0.15mm, a working pressure of 0.1 KPa, a drip-head flow rate of 2L h^-1^, and a drip-head spacing of 50cm in such a way that one drip-head is serving one potato plant. Each plot had an independent unit of irrigation control system, including a water meter, gate valve and pressure gauge, etc. Crops were fertilized based on the fertilizer recommendations for potato with Urea (46% nitrogen, 210 kg ha^-1^), Calcium superphosphate (26% phosphorus, 82.5 kg ha^-1^) and Potassium sulfate (50% potassium, 150 kg ha^-1^). The nitrogen fertilizer was applied as split application with water at six times during the seedling stage, tuber formation stage, tuber growth stage and starch accumulation stage at the ratio of 1:2:2:1, respectively. Other crop management practices such as weeding and crop protection were carried out in accordance with the recommended agronomic practices in the region. The potato crop was harvested on September 27 at both years of the experiment.

### 2.3 Crop measurements

#### 2.3.1 Leaf area index

Leaf area was measured at all growth stages of potato using the LI-3000C portable leaf area meter (LI-COR Biosciences, Lincoln, NE, United States). The leaf area index was calculated as the ratio of total leaf area to the land area (Yan et al., 2021).

#### 2.3.2 Canopy cover

Canopy cover is an important indicator of the crop growth process simulated by the AquaCrop model and is calculated as follows (Hsiao et al., 2009).


(1) $$CC=1\text{.005[1-exp(-0.6}LAI{\text{)]}}^{1.2}$$

where *CC* is canopy coverage and *LAI* is the leaf area index.

#### 2.3.3 Above-ground biomass

Three representative plants were randomly selected from each plot at each growth stage of potato, the roots, stems, leaves and tubers were separated and washed with water. The water on the plant samples was blotted by filter paper and the samples were placed in archive bags. These samples were oven dried at 105°C for 30 minutes and then dried at 75°C to a constant weight. The biomass content of each plant part was determined using an electronic balance after cooling to the room temperature (Högy and Fangmeier, 2009).

#### 2.3.4 Tuber yield

An area of 2 x 2 m of potato crops were randomly selected from each treatment plot, weighed for individual yield, then the average yield per plant was calculated. The total yield per hectare of potato for each treatment was then calculated according to the planting density.

#### 2.3.5 Water use efficiency

Water use efficiency (WUE) is the amount yield produced per unit of water consumed, and WUE was calculated using the following equation (Ali and Talukder, 2008).


(2) $$WUE=\frac{Y}{ET}$$

where *Y* is yield (t ha^-1^) and *ET* is the water consumption of potatoes as crop evapotranspiration (mm).

### 2.4 Principles of AquaCrop modeling

The AquaCrop model takes days as the simulation step, and it can simulate the processes namely, soil water balance, crop growth and development, crop transpiration, aboveground dry matter production, and final yield formation. The AquaCrop model mainly focuses on the response of crop growth and development to water, and its core formula for yield *Y* is calculated as follows.


(3) $$Y=f_{HI}HI_{0}B\;$$

where *f_HI_* is the adjustment factor, which can be affected by the effects of stresses such as water stress and temperature stress at the time of yield formation and at the time of crop pollination; *HI_0_* is the reference harvest index, and *B* is the final above-ground biomass at harvest (Vanuytrecht et al., 2014).

In the absence of cold temperature stress, the above-ground biomass B can be calculated from crop’s transpiration (*Tr*) using the following equation (Vanuytrecht et al., 2014).


(4) $$B=WP^{*}\sum \frac{T_{r}}{ET_{0}}$$

Where WP* is the normalized biomass water productivity, *Tr* is the crop’s transpiration and *ET_0_* is the reference evapotranspiration.

The crop’s transpiration *Tr* was calculated as follows.


(5) $$T_{r}=K_{S}\left(CC^{*}K_{C_{Tr,x}}\right)ET_{0}$$

where *Ks* is the water stress coefficient (i.e., drought or water logging), CC* is the adjusted green canopy cover and *K_CTr, x_* is the maximum crop transpiration coefficient (Raes et al., 2009; Vanuytrecht et al., 2014). The version of AquaCrop model used in this study was Version 6.0.

### 2.5 Data inputs for the AquaCrop model

#### 2.5.1 Meteorological data

The field meteorological data for the period of 2015 – 2020 were obtained from the China Meteorological Data Network (Concentric site: 53810) that consists of maximum temperature, minimum temperature, precipitation, sunshine hours, relative humidity and average wind speed at daily time scale. The reference crop evapotranspiration (*ET_0_*) was calculated according to the Penmen-Monteith formula (López-Urrea et al., 2006), as follows.


(6) $$ET_{0}=\frac{0.408\Delta \left(R_{n}-G\right)+\gamma \frac{900u_{2}}{T+273}\left(e_{s}-e_{a}\right)}{\Delta +\gamma \left(1+0.34u_{2}\right)}$$

where *ET_0_* is reference evapotranspiration (mm day^-1^); *Rn* is net radiation at the crop surface (MJ m^-2^ day^-1^); *G* is soil heat flux density (MJ m^-2^ day^-1^); *T* is mean daily air temperature at 2 m height (°C); *u_2_* is wind speed at 2 m height (m s^-1^); *es* is saturation vapour pressure (kPa); *ea* is actual vapour pressure (kPa); *es-ea* is saturation vapour pressure deficit (kPa); Δ is slope vapour pressure curve (kPa °C^-1^); *γ* is psychrometric constant (kPa C^-1^).

**Figure 2:**
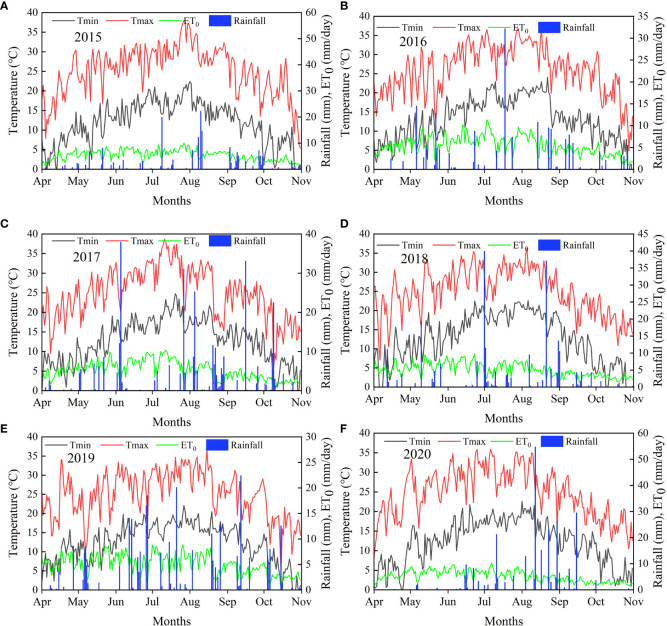
Daily rainfall, minimum temperature (Tmin), maximum temperature (Tmax), and evapotranspiration (ET_0_) during the period of 2015 – 2020 at the experimental site. Figures **(A–F)** show meteorological data for 2015, 2016, 2017, 2018, 2019, and 2020, respectively.

The daily rainfall, maximum and minimum temperatures and *ET_0_* from 2015 to 2020 are presented in **Figure 2**. The rainfall from May-October during the planting phase of the experiment was 234.2 mm and 266.6 mm in 2019 and 2020, respectively.

#### 2.5.2 Soil data

Soil parameters of AquaCrop model mainly consist of soil compartment number, compartment thickness (m) and associated soil layer number (Vanuytrecht et al., 2014). Before planting the crops, soil samples were taken from five randomly selected plots in the field at different depths, and required soil parameters were measured (**Table 3**) and imported into the model to create the soil data file (SOL).

**Table 3:** Measured soil parameters at different depths of the experimental location.

Soil depth (cm)	Wilting point (cm^3^/cm^3^)	Field capacity (cm^3^/cm^3^)	Saturated water content (cm^3^/cm^3^)	Bulk density (g/cm^3^)	Clay %	Silt %	Sand %
0-20	0.10	0.19	0.32	1.41	21.28	5.72	73.00
20-40	0.10	0.20	0.35	1.38	22.89	4.21	72.90
40-60	0.12	0.23	0.38	1.36	22.29	4.01	73.70

#### 2.5.3 Crop data

The crop parameter file consists of crop growth, crop evapotranspiration, crop production and moisture, salinity, and temperature stresses. The initial and maximum canopy cover, days taken to flowering, senescence and maturity were obtained from actual field observations, while the water productivity, harvest index, moisture and temperature stress response coefficients of crop production were determined based on the baseline parameters provided by the model. CO_2_ concentration was derived from the default data recommended by the model namely A1B.CO_2_, with correction by the ‘trial and error method’. The main model parameters of the calibrated AquaCrop model are presented in **Table 4**.

**Table 4:** Model parameters of the calibrated AquaCrop model.

Parameter	Definition	Calibrated value	Unit
T_base_	Base temperature	10	°C
T_upper_	Upper limit temperature	35	°C
K_cTR_	Crop coefficient	1.15	
CC_o_	Initial canopy cover	0.9	%
CGC	Canopy growth coefficient	0.966	%/d
CC_x_	Maximum canopy cover	94	%
CDC	Canopy decay coefficient	0.798	%/GDD
WP	Standard water productivity	20	g·m^-2^
	Maximum effective root depth	0.45	m
HI_0_	Reference harvest index	55	%
P_exp,upper_	Upper limit of the effect of water stress on canopy	0.26	NA
P_exp,lower_	Lower limit of the effect of water stress on canopy	0.66	NA
P_clo,upper_	Upper limit of the effect of water stress on stomatal conductance	0.65	NA
P_sen,upper_	Upper limit of the effect of water stress on early canopy senescence	0.69	NA

NA, not applicable.

### 2.6 Crop model evaluation

To verify the goodness of fit of the simulated values with the measured values, the root mean square error (*RMSE*), the index of agreement (*d*), the normalized root mean square error (*NRMSE*) and the coefficient of determination (*R^2^*) were used as model evaluation parameters (Eeswaran et al., 2021).


(7) $$RMSE=\sqrt{\sum_{i=1}^{n}\frac{{{\text{(S}}_{\text{i}}{\text{-M}}_{\text{i}})}^{2}}{\text{n}}}$$

(8) $$NRMSE=\sqrt{\sum_{\text{i}=1}^{\text{n}}\frac{{\left({\text{S}}_{\text{i}}-{\text{M}}_{\text{i}}\right)}^{2}}{n}}\times 100/\bar{\text{M}}$$

(9) $$d=1-\frac{\sum_{\text{i}=1}^{\text{n}}{\left({\text{S}}_{\text{i}}-{\text{M}}_{\text{i}}\right)}^{2}}{\sum_{\text{i}=1}^{\text{n}}{\left(\left|{\text{S}}_{\text{i}}-\bar{\text{M}}\right|+{\text{M}}_{\text{i}}-\bar{\text{M}}\right)}^{2}}$$

(10) $$R^{2}=\frac{{\left[\sum_{i=1}^{n}\left({\text{S}}_{\text{i}}-\bar{\text{S}}\right)\left({\text{M}}_{\text{i}}-\bar{\text{M}}\right)\right]}^{2}}{\sum_{\text{i}=1}^{\text{n}}{\left({\text{S}}_{\text{i}}-\bar{\text{S}}\right)}^{2}\sum_{\text{i}=1}^{\text{n}}{\left({\text{M}}_{\text{i}}-\bar{\text{M}}\right)}^{2}}$$

where, *Si* is the simulated value, *Mi* is the measured value, *n* is the number of measurement samples, and $\bar{M}\;$ is the average of the measured values. When the calculated values of *RMSE* and *NRMSE* are smaller, the more accurate the simulation results will be. When the value of *d* is close to 1, the better the fit between the simulation results and the measured results and, when the value of *R^2^* is close to 1, the model is reliable, and when the value of *R^2^* is close to 0, the simulation results are average.

### 2.7 Simulation of different irrigation scenarios

To investigate the effects of different irrigation scenarios (i.e., based on the field experiment) on yield and water use efficiency of potato under the drip irrigation, two different irrigation simulation scenarios were developed based on meteorological data for a period of 6 years from 2015 to 2020. The details of these simulation scenarios are presented in **Table 5**. Yield, biomass, water use efficiency and water consumption were simulated under both of these simulation scenarios.

**Table 5:** Details of the simulation scenarios.

	Simulation scenario	Irrigation amount (mm)	Irrigation cycle (days)	Number of irrigation events	Total irrigation volume (m^3^/ha)		Simulation scenario	Irrigation quota/irrigation event (mm)	Irrigation cycle (days)	Number of irrigation events	Total irrigation volume (m^3^/ha)
Simulation scenario I	A1	90	5	12	900	Simulation scenario II	A16	10	5	12	1200
	A2	90	7	9	900		A17	10	7	9	900
	A3	90	10	6	900		A18	10	10	6	600
	A4	120	5	12	1200		A19	13	5	12	1560
	A5	120	7	9	1200		A20	13	7	9	1170
	A6	120	10	6	1200		A21	13	10	6	780
	A7	150	5	12	1500		A22	18	5	12	2160
	A8	150	7	9	1500		A23	18	7	9	1620
	A9	150	10	6	1500		A24	18	10	6	1080
	A10	180	5	12	1800		A25	20	5	12	2400
	A11	180	7	9	1800		A26	20	7	9	1800
	A12	180	10	6	1800		A27	20	10	6	1200
	A13	210	5	12	2100		A28	24	5	12	2880
	A14	210	7	9	2100		A29	24	7	9	2160
	A15	210	10	6	2100		A30	24	10	6	1440

A1 - A30 are the different simulation scenarios.

The irrigation amount is the sum of the successive irrigations for the entire crop reproductive period. The irrigation quota is the amount of water that can be filled at an irrigation event. The irrigation cycle is the maximum time interval between two successive irrigations to meet the water requirements of the crop under the conditions of defined irrigation quota and daily water consumption.

## 3 Results

### 3.1 Calibration of AquaCrop model

**Figure 3:**
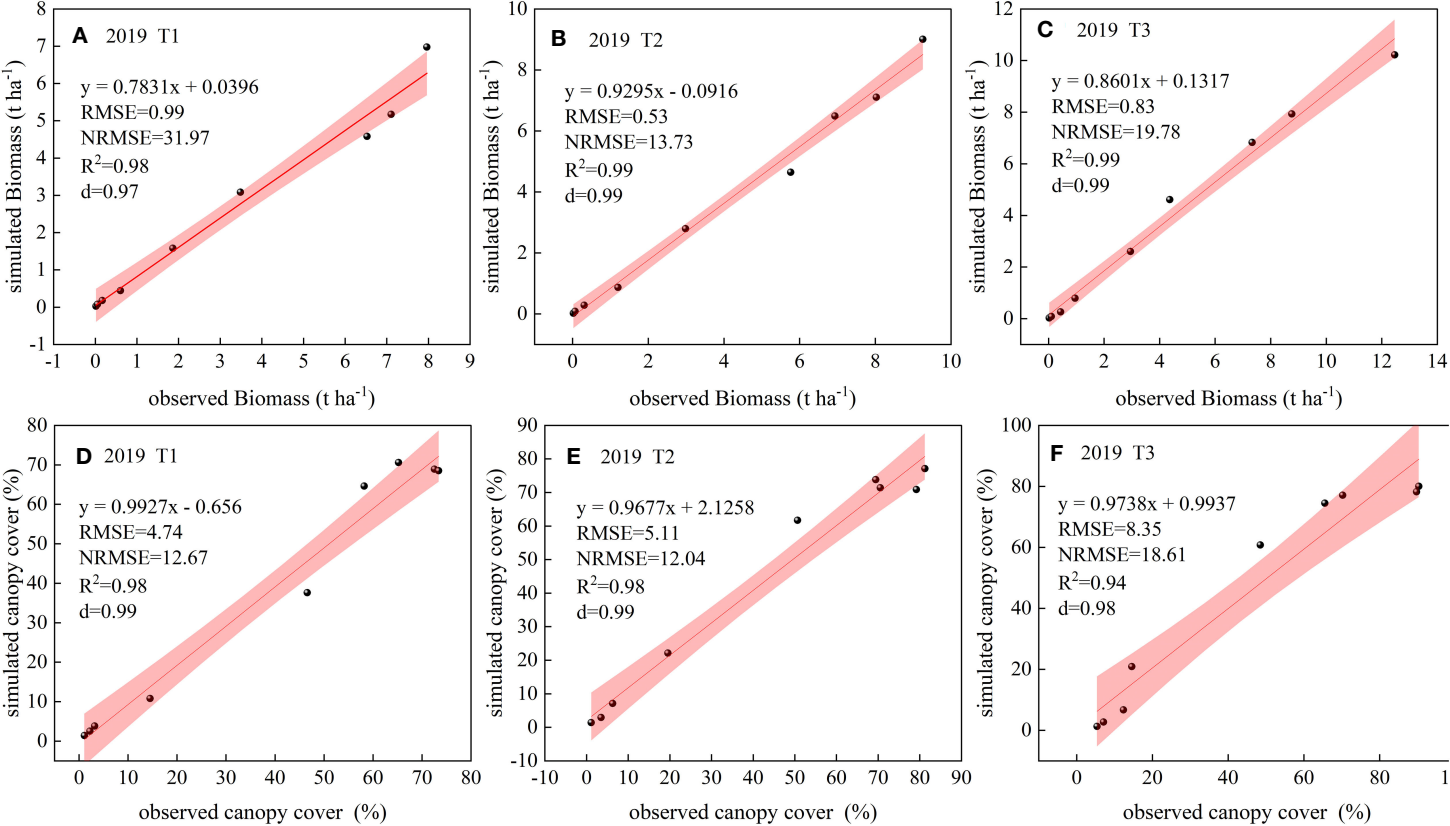
Comparisons of observed and simulated values of biomass **(A–C)** and canopy cover **(D–F)** at model calibration with the respective model evaluation parameters.

The AquaCrop model was calibrated using the 2019 experimental data for biomass and canopy cover for treatments T1, T2 and T3. The values obtained for the model evaluation parameters are presented in **Figure 3**. The *RMSE* values for biomass and canopy cover were less than 10%, indicating very good calibration of the model parameters. Moreover, the NRMSE values for canopy cover and biomass showed moderate (<30%) goodness of fit. As per the index of agreement (*d*) and the coefficient of determination (*R^2^*), excellent (>0.9) goodness of fit was observed between simulated and observed biomass and canopy cover (Eeswaran et al., 2021). These results indicate that the simulated values of the AquaCrop model fit well with the observed values, and the calibrated model parameters can be used to simulate the growth and development process of potato under the drip irrigation in the arid zone of Ningxia.

### 3.2 Validation of AquaCrop model

**Figure 4:**
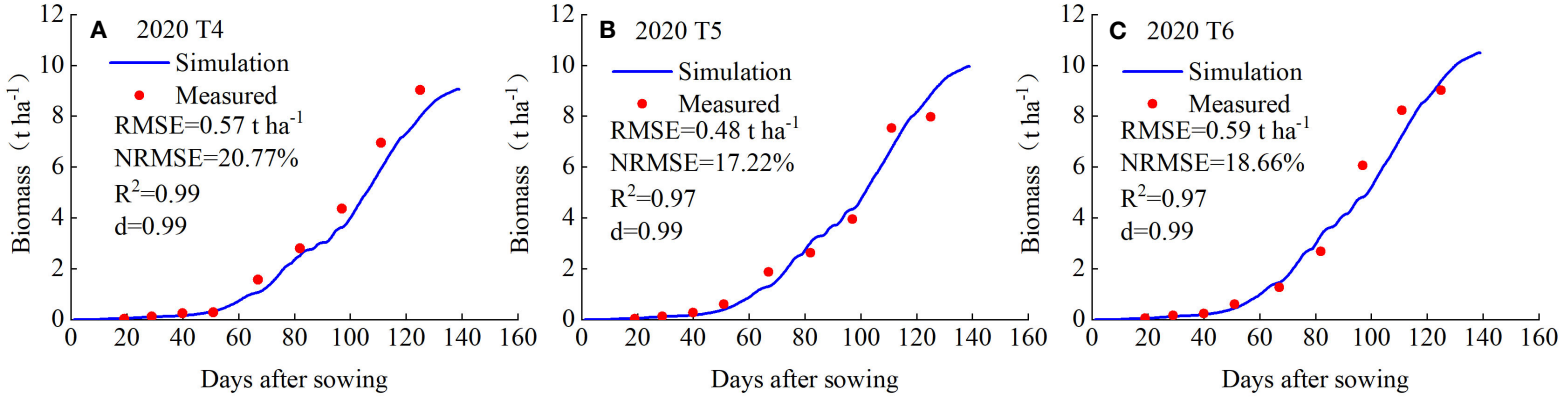
Measured and simulated values of potato biomass for the treatments T4 **(A)**, T5 **(B)** and T6 **(C)** in 2020.

**Figure 5:**
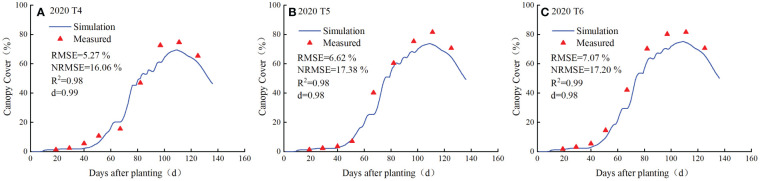
Measured and simulated values of canopy cover of potato for the treatments T4 **(A)**, T5 **(B)** and T6 **(C)** in 2020.

The calibrated AquaCrop model was validated using the measured data from T4-T6 irrigation treatments in 2020, and the validation results are shown in **Figures 4**, **5**. Accordingly, the canopy cover was small until 50 days after planting (DAP) and increased rapidly as the potatoes entered the rapid growth phase, and the crop reached the maximum canopy cover at around 110 DAP.

As shown in **Figure 4**, the simulated biomass was consistent with the measured biomass in all three treatments. Potato biomass began to accumulate slowly 20 DAP and entered the rapid growth phase at about 50-60 DAP, the growth rate of potato biomass increased and reached its maximum at about 140 DAP and the growth was ceased thereafter. The goodness of fit indicators (*RMSE*, *NRMSE*, *R^2^* and *d*) highlight that the simulated canopy cover is representative to the measured values under all the treatments (**Figure 5**). Therefore, the model was reasonably calibrated and validated for the simulation of the growth of potato.

**Figure 6:**
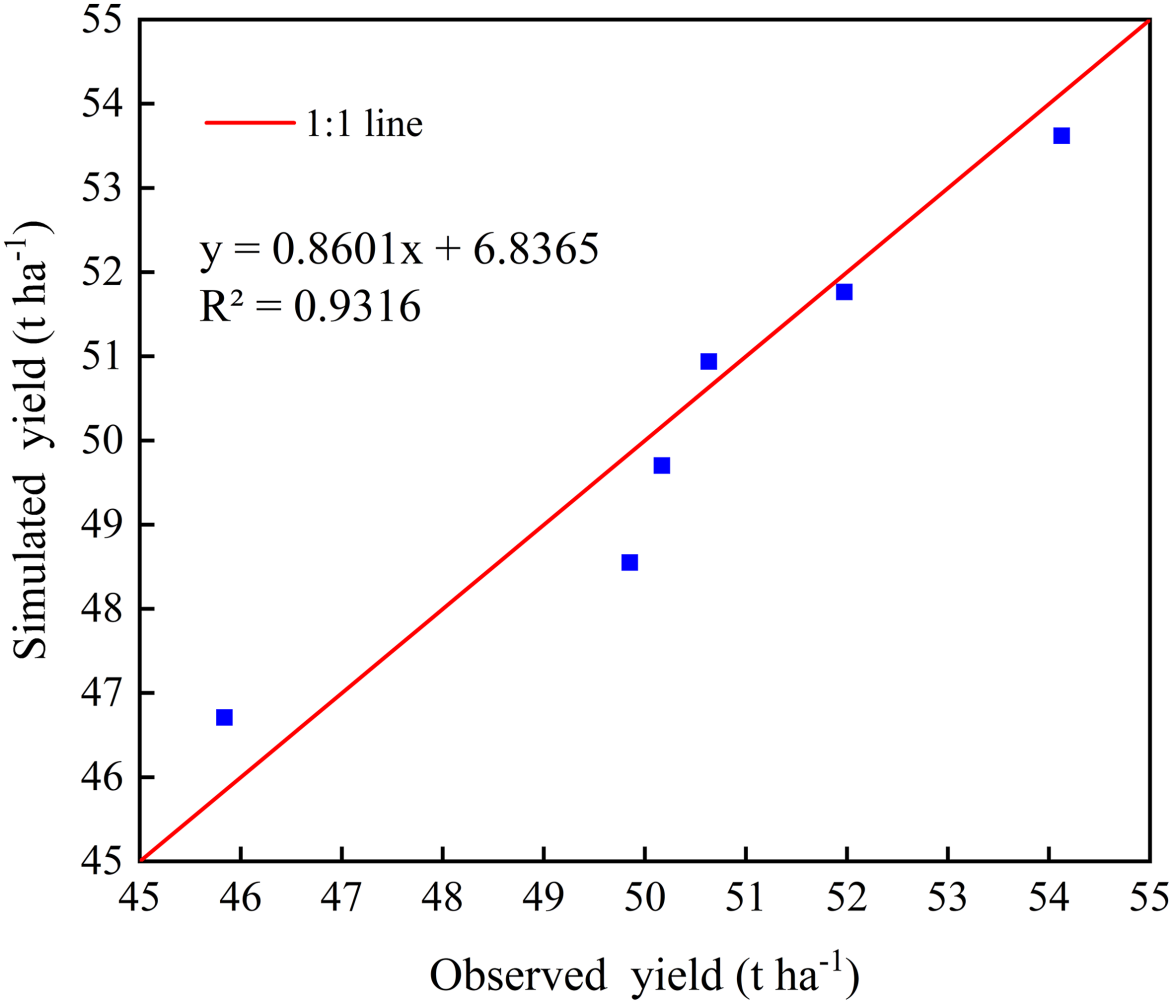
Comparison between measured and simulated tuber yield of potato under all treatments in 2019-2020. The solid red line indicates the line of 1:1 perfect agreement.

The comparison between the simulated and measured tuber yield of potato when all the treatment results are pooled is presented in **Figure 6**. Accordingly, the coefficient of determination (*R^2^*) obtained was 0.93, highlighting the greater goodness of fit between simulated and measured tuber yield of potatoes.

### 3.3 Optimization of irrigation for potatoes grown under drip irrigation

The calibrated and validated AquaCrop model was used to simulate yield and biomass under different irrigation scenarios by importing six years of meteorological data from 2015-2020 and the multi-year averages were calculated. The irrigation cycle is the frequency of irrigation and refers to the longest time interval between irrigations under the condition that the irrigation amount and daily water consumption can meet the water requirements of the crop. Fifteen scenarios with different irrigation frequencies under fixed irrigation amounts were initially simulated (**Table 6**). Accordingly, tuber yield increased with decreasing frequency at 90 and 120 mm of irrigation, increased with increasing frequency at 150 and 180 mm of irrigation, and increased and then decreased with increasing frequency at 210 mm of irrigation. The maximum tuber yield (43.40 t ha^-1^) was achieved with the A10 irrigation scheme, and the minimum yield (27.89 t ha^-1^) was achieved under the A1 scheme. The water use efficiency of potato showed an increasing trend with decreasing irrigation frequency for 90, 120, 150 and 210 mm of irrigation, and a decreasing trend with decreasing irrigation frequency for 180 mm of irrigation, with a maximum water use efficiency being 12.97 kg m^-3^ under the A10 scheme. Therefore, the A10 scheme increases both the tuber yield and water use efficiency under simulation scenario I (**Table 6**).

**Table 6:** Tuber yield, biomass, water use efficiency (WUE) and evapotranspiration (ET) of potato under simulation scenario I.

Simulation scheme	Irrigation amounts (mm)	Irrigation cycle (day)	Number of irrigation events	Yield (t ha^-1^)	Biomass (t ha^-1^)	WUE (kg m^-3^)	ET (mm)
A1	90	5	12	27.89	7.13	10.50	260
A2	90	7	9	28.00	6.79	10.71	254
A3	90	10	6	28.30	6.56	11.37	242
A4	120	5	12	30.25	7.70	10.65	279
A5	120	7	9	30.45	7.63	10.86	273
A6	120	10	6	31.00	7.22	11.73	258
A7	150	5	12	34.33	8.62	11.16	303
A8	150	7	9	33.50	8.34	11.22	291
A9	150	10	6	33.03	7.61	12.01	269
A10	180	5	12	43.40	9.62	12.97	332
A11	180	7	9	40.88	9.16	12.81	314
A12	180	10	6	34.47	8.1	11.99	281
A13	210	5	12	36.99	9.09	11.55	318
A14	210	7	9	37.28	8.77	12.12	301
A15	210	10	6	34.26	7.92	12.14	276

The simulation of 15 scenarios with different irrigation frequencies at fixed irrigation quota in **Table 7** shows that the yield increases with decreasing irrigation frequency at the same irrigation quota, with the highest yield (49.76 t ha^-1^) and biomass (10.53 t ha^-1^) at an irrigation quota of 20 mm and 5 days irrigation cycle in scenario A25. The water use efficiency tends to decrease and then increase with decreasing irrigation frequency at 10, 13 and 18 mm of irrigation quota, and decreases with decreasing irrigation frequency at 24 mm of irrigation quota, while it increases and then decreases at 20 mm of irrigation quota The highest water use efficiency (15.74 kg m^-3^) and yield of 46.77 t ha^-1^ obtained at 7 days of irrigation cycle under the simulation scheme A26.

**Table 7:** Tuber yield, biomass, ET and water use efficiency (WUE) and evapotranspiration (ET) of potato under simulation scenario II.

Simulation scheme	Irrigation quota (mm)	Irrigation cycle (day)	Number of irrigation events	Yield (t ha^-1^)	Biomass (t ha^-1^)	WUE (kg m^-3^)	ET (mm)
A16	10	5	12	30.25	7.70	10.65	279
A17	10	7	9	28	6.79	10.61	256
A18	10	10	6	25.28	5.63	11.04	223
A19	13	5	12	34.33	8.62	11.16	303
A20	13	7	9	30.45	7.52	10.86	273
A21	13	10	6	27.03	6.22	11.14	236
A22	18	5	12	43.4	9.62	12.97	332
A23	18	7	9	34.69	8.49	11.47	295
A24	18	10	6	30.00	7.02	11.58	253
A25	20	5	12	49.76	10.53	13.74	358
A26	20	7	9	46.77	9.28	15.74	300
A27	20	10	6	32.62	7.81	11.95	267
A28	24	5	12	44.01	9.71	13.01	333
A29	24	7	9	38.40	8.98	12.18	311
A30	24	10	6	30.81	7.22	11.69	258

**Figure 7:**
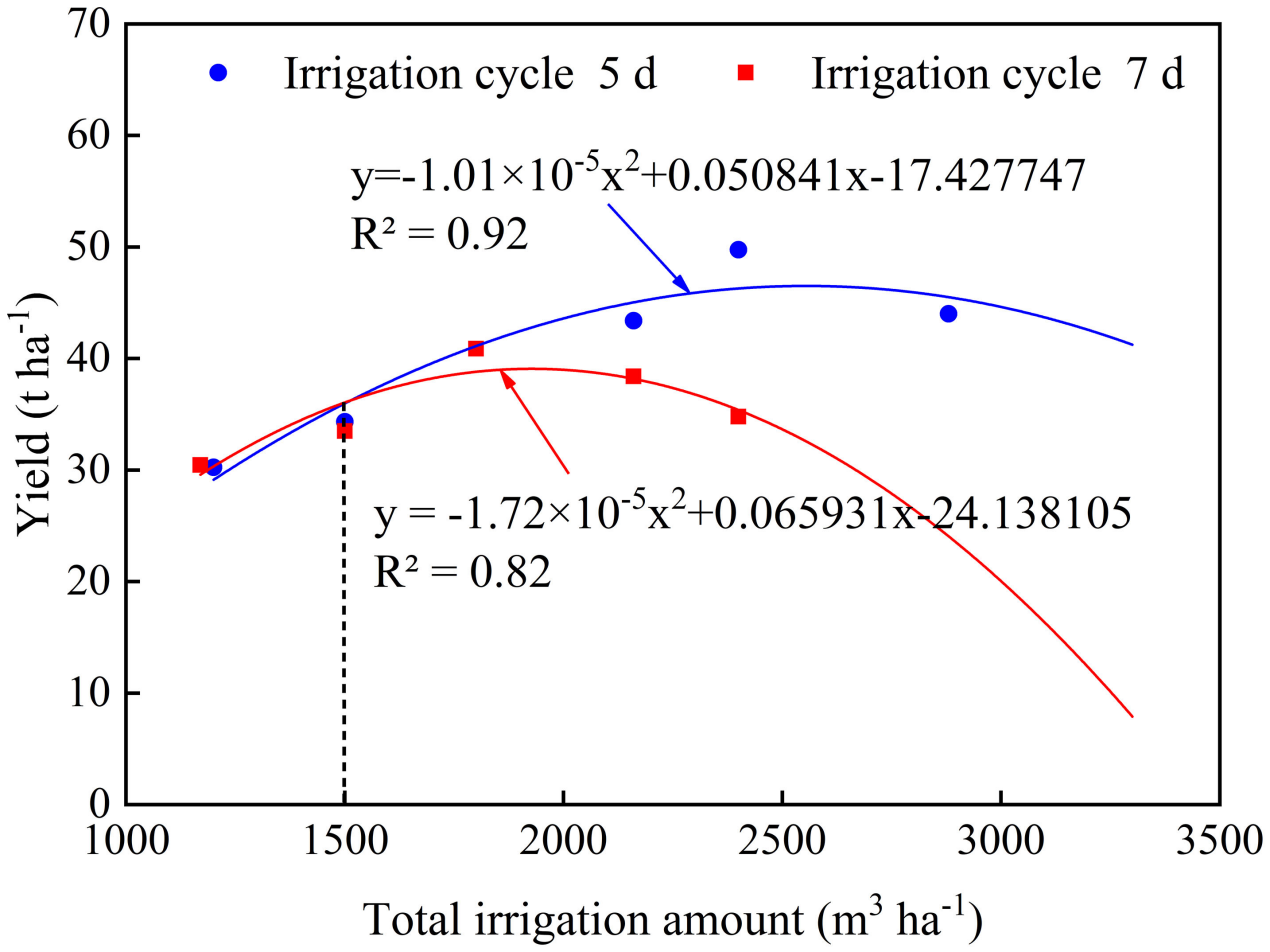
The relationship curve between total irrigation applied and potato yield under different irrigation cycles.

As the yield was lower with the irrigation cycle at 10 days, we analyzed the data only from the 5 days and 7 days irrigation cycles. Accordingly, the irrigation cycle at 7 days yielded higher when the total irrigation volume was less than 1500 m^3^ ha^-1^, and the irrigation cycle at 5 days yielded more when the total irrigation volume was greater than 1500 m^3^ ha^-1^. Overall, the potato yield increased and then decreased with increasing irrigation, and the fitted results followed a quadratic equation (Zheng et al., 2013), with *R^2^* of 0.92 for a 5 days irrigation cycle. The *R^2^* was slightly decreased (0.82) for a 7 days irrigation period, with a more pronounced decline in yield with increasing amount of irrigation (**Figure 7**). Without considering the workload and cost for water resources in the field, the maximum yield (49.76 t ha^-1^) was achieved from the A25 scheme with 5 days irrigation cycle and 2400 m^3^ ha^-1^ of total water application. However, when water economy and field work are considered together, the A26 scheme has produced the optimum yield (46.77 t ha^-1^).

These results provide insights into the growth and yield response of potatoes to different irrigation levels under the drip irrigation thus provide an opportunity to optimize the irrigation recommendations. The implications and utility of these findings are discussed below.

## 4 Discussion

### 4.1 Applicability of the AquaCrop model to simulate growth and yield of potato

Although conclusions obtained from field experiments are accurate and reliable, field trials are limited by geographical, climatic, site, financial and human factors (Martins et al., 2018). In this study, two years of field experimental data were used to calibrate and validate the growth and yield of potato under drip irrigation in the arid zone of Ningxia using the AquaCrop model. The values of *RMSE*, *NRMSE*, *d* and *R^2^* for the comparison of simulated *vs.* observed values of crop biomass, canopy cover and yield have shown the ability of the calibrated AquaCrop model to simulate growth and yield of potato in response to water. Similarly, Rankine et al. (2015) showed that the AquaCrop model can simulate growth and yield of sweet potato under temperate conditions and can be a very useful tool for decision support on irrigation timing and volume. Therefore, this AquaCrop model has reasonable applicability to simulate the growth and yield of potato under the drip irrigation in the arid zone of Ningxia and can provide a theoretical basis for the local potato simulation studies.

### 4.2 Optimization of different irrigation scenarios for potatoes under the drip irrigation

Crop models have become the most beneficial tools to optimize crop management (Holzworth et al., 2014). Potato production in the central arid zone of Ningxia is substantially affected by water scarcity. Hence, different irrigation scenarios were simulated the calibrated AquaCrop model for potato irrigation fixation with varying irrigation amounts and irrigation cycles. In this paper, we initially simulated 15 scenarios in which the irrigation amount was fixed, and the number of irrigation cycles was changed. The results show that the yield decreases as the irrigation amount decreases, with the highest yield at 180 mm of irrigation at a 5-day irrigation cycle. These results are consistent with those of previous studies (Lehmann and Finger, 2014; Li et al., 2022) where it has been shown that decreasing the irrigation decreased the potato yields, but one simulation study has shown that excessive irrigation resulted in lower yields following the water stress (Jama-Rodzenska et al., 2021). Our second set of simulations of 15 scenarios with fixed irrigation quota and varying irrigation cycles showed that a 20 mm irrigation quota at a 5 -day irrigation cycle resulted in the highest tuber yield than all the other scenarios. The lower yields were observed with lower irrigation amounts and quotas probably due to the moisture stress as the depth of wetting was not reaching the effective root zone of the potato crop and substantially reducing the canopy cover and crop growth (Yuan et al., 2003; Mattar et al., 2021).

### 4.3 Recommendation of irrigation for potatoes under the drip irrigation

To improve irrigation and water use efficiency, it is important to optimize scheduling of irrigation (Li et al., 2019). Accordingly, A10 scheme of 180 mm maximum of water produced higher yield in simulation scenario I, which is consistent with the maximum yield observed in a previous study with 1800 m^3^ ha^-1^ of water at the same level of nitrogen application (Yin et al., 2020).

However, changing the frequency of irrigation (i.e., irrigation cycle) can affect the potato yield (Wang et al., 2006) as the irrigation frequencies can modify plant physiological processes, growth, yield and water use efficiency (Boyle et al., 2016). At the same irrigation frequency, increasing the irrigation quota could increase the biomass (Si et al., 2020), which was similar to the study of Su et al. (2022) on the linear increase of crop biomass with irrigation quota at a certain irrigation range. Furthermore, irrigation scheduling is an important factor affecting water use efficiency, and water-saving irrigation facilitates the conservative use of irrigation water (Li et al., 2020). The analysis of irrigation optimization (**Figure 7**) showed that A26 scheme (1800 m^3^ ha^-1^ of total irrigation at 7 days irrigation cycle) has produced the optimum yield with highest water use efficiency. Although A25 scheme with 5 days irrigation cycle and 2400 m^3^ ha^-1^ of total irrigation has produced the highest yield, 600 m^3^ ha^-1^ of additional irrigation only improved the yield by 6.4% thus reducing the water use efficiency of the crop. This was because increasing irrigation frequency increased potato yield but failed to increase the water use efficiency in potato as shown by Liu et al. (2019). Therefore, 20 mm of irrigation quota at 7 days of irrigation cycle can be considered as an optimum irrigation scheduling for potato cultivated under the drip irrigation in the arid regions. Nevertheless, the optimum irrigation system should be adjusted considering the local climate, fertilizer application and crop variety (Zheng et al., 2013). These findings can provide the baseline for rational water and fertilizer management for local potato production in the arid regions such as Ningxia in China.

## 5 Conclusions

The objective of this study was to optimize the irrigation for potato cultivated under the drip irrigation in the arid region of Ningxia, China. The AquaCrop model was calibrated and validated using the data from two years of field experiment in devising the optimal irrigation recommendation. The calibrated crop model was used to simulate the changes in canopy coverage, biomass and tuber yield of potato with 30 different irrigation schemes under two different irrigation scenarios. The results showed that the AquaCrop model could better simulate the growth and yield of potato under the drip irrigation with different irrigation treatments. We found that increasing the total amount of irrigation could increase the yield of potato under the drip irrigation; however, yield begins to decline when the amount of total irrigation exceeds 2500 m^3^ ha^-1^. Moreover, the optimum irrigation schedule for potatoes was 20 mm of irrigation quota at 7 days of irrigation cycle which requires 1800 m^3^ (meter cube) ha^-1^ of total irrigation. The above irrigation scheduling has achieved 46.77 t ha^-1^ of tuber yield with 15.74 kg m^-3^ of water use efficiency. Future research should focus on evaluating the best performing irrigation schemes at different climate and soil conditions for potato cultivation in various parts of the arid regions.

## Data availability statement

The original contributions presented in the study are included in the article/supplementary material. Further inquiries can be directed to the corresponding authors.

## Author contributions

Conceptualization, JY and YY. Methodology, JY, YY and RE. Open field experiments and data compilation, YY. Software, YY. Validation, YY. Formal analysis, JY, YY. Resources, JY, YY. Data curation, RE, ZM, ZY and FS. Writing—original draft preparation, YY, ZM, ZY and FS. Writing—review and editing, YY and RE. Visualization, YY. All authors contributed to the article and approved the submitted version.
